# Quantifying bite force in coexisting tayassuids and feral suids: a comparison between morphometric functional proxies and *in vivo* measurements

**DOI:** 10.7717/peerj.11948

**Published:** 2021-08-12

**Authors:** Fernando L. Sicuro, Luiz Flamarion B. Oliveira, Carla D. Hendges, Carlos Fonseca

**Affiliations:** 1 CESAM—Centre for Environmental and Marine Studies, Department of Biology, Universidade de Aveiro, Aveiro, Portugal; 2 IBRAG—Instituto de Biologia Roberto Alcântara Gomes, Universidade do Estado do Rio de Janeiro, Rio de Janeiro, Rio de Janeiro, Brazil; 3 Museu Nacional, Departamento de Vertebrados, Universidade Federal do Rio de Janeiro, Rio de Janeiro, Brazil; 4 Departamento de Ciências da Narureza, Universidade Comunitária da Região de Chapecó, Chapecó, Santa Catarina, Brazil; 5 ForestWISE, Laboratório Colaborativo Para a Gestão Integrada da Floresta e do Fogo, Vila Real, Portugal

**Keywords:** Tayassuidae, Feral Suidae, Ecomorphology, Functional morphology, *In vivo* bite measurement, Bite force

## Abstract

**Background:**

Measuring mammals’ bite force in laboratory conditions is not a simple task, let alone on wild medium-sized mammals in the field. Thus, morphometric-proxies are usually used to infer morphofunctional properties of musculoskeletal features. For instance, the study of bite force-indexes suggests that different capacities to crack food items reduce the competition between coexistent collared and white-lipped peccaries (*Pecari tajacu* and *Tayassu pecari*). The presence of exotic feral hogs (*Sus scrofa*) in peccaries’ endemic areas gives rise to new ecological interactions between them. An example is the Brazilian Pantanal wetland, where ecomorphological mechanisms may play a role in their ecological relations. Taking this scenario as a case of study, we aimed to verify if the morphometric-proxies are *de facto* reliable tools, by comparing bite forces-indexes with the *in vivo* bite forces of these species.

**Methods:**

We captured 21 collared and white-lipped peccaries and feral hogs in the Brazilian Pantanal to assess their bite force at first molar. The Bite Force Measuring Tube (BiTu) is a robust and simple mechanical device designed to be used in field conditions. Only 11 individuals successfully bit the BiTu before being released. Their body measurements were compared and correlated with their bite force. The *in vivo* bite forces were compared with bite force-indexes of two papers based on independent morphometric methods and datasets: Sicuro & Oliveira (2002) used classic morphometrics to infer the bite forces of these three species in the Brazilian Pantanal, and Hendges et al. (2019) used geometric morphometrics to compare bite forces-indexes and feeding habits of the extant peccary species. The results of all species were standardized (Z-curves) according to each method. Doing so, we obtained comparable dimensionless comparable values but maintaining the differences between them.

**Results:**

The morphometric-proxies-based studies presented similar results: collared peccaries present weaker bites than white-lipped peccaries and feral hogs, while these two species presented no significant differences in their bite force-indexes. The *in vivo* bite force results suggest the same relations predicted by the morphometric models, including the high variation among the feral hogs. We found a significant correlation between the individuals’ weight (kg) and their actual bite force (*N*) but no significant correlations with the head length.

**Conclusions:**

The BiTu proved to be a functional and low-cost tool to measure bite force in field conditions. The *in vivo* results presented a good correspondence with the predictions based on morphometric-proxies by Sicuro & Oliveira (2002) and Hendges et al. (2019). The results denote that these studies succeed in capturing the biomechanical signal of the three species’ skull-jaw systems. This empirical validation confirms that these morphometric-proxies analyses are reliable methods to ecomorphological and evolutionary inferences.

## Introduction

The idea that contraction force was related to dimensions and weight of muscle groups dates back to the first half of the XXth century with the studies of Bethe (1923) and Becht (1953). In his classical book about vertebrate functional morphology and structure, Hildebrand (1974) has postulated that the maximum force of a muscle is equal to each muscle fiber’s contraction force, multiplied by the total number of fibers. Therefore, the overall force of a given muscle is proportional to this muscle’s cross-sectional area. Nevertheless, Hildebrand (1974) points out that the validity of his statement relies on three assumptions: (i) muscle fibers must be parallel with each other; (ii) they must have the same length; and (iii) all muscle fibers must contract at the same time and equally. Although these conditions are rarely met, this principle is almost a biomechanical axiom and, in many ways, it is used in most functional modeling. This approach has been the primary way to understand ecomorphological and morphofunctional evolutionary relationships between phylogenetically and/or morphologically related groups (Ricklefs & Miles, 1994).

Much of what is known about mammalian biomechanics relies on the assumption that function can be inferred from anatomical traits or bones’ structural properties. Measurements of muscle scars and lever systems based on bone structures have been used as proxies to infer properties of skull-jaw mechanics and make functional comparisons among mammalian groups (Davis, 1955; Davis, 1964; Turnbull, 1970; Kiltie, 1982; Kiltie, 1985; Emerson & Radinsky, 1980; Radinsky, 1981a; Radinsky, 1981b; Radinsky, 1987; Sicuro & Oliveira, 2002; Therrien, Henderson & Ruff, 2005; Wroe, McHenry & Thomason, 2005; Sicuro & Oliveira, 2011; Hendges et al., 2019). In the last two decades, Finite Element Analysis (FEA) became a new way to assess stress resistance of osteological structures to strain forces, such as compression, shearing, and torsion (Richmond et al., 2005; Fletcher, Janis & Rayfield, 2010; Tseng & Binder, 2010; Tseng et al., 2011; Cox, Rinderknecht & Blanco, 2015; Therrien et al., 2016; Lautenschlager et al., 2017; Morales-García et al., 2019). Some of these methods are quite complex and Cardini (2020) criticizes what he considers an excessive focus on complex mathematical models to describe the form and function of structures rather than their real ecomorphological meaning. However, from simple 2D models to complex FEA or high-density morphometric models, there is a common hindrance: assessing the actual magnitude of the forces involved in the biomechanical systems (*i.e.,* in *N* units of force). Therefore, knowing the forces involved in craniomandibular systems is a piece of valuable information to support biomechanical evolutionary studies and ecomorphological propositions. The assessment of the empirical magnitude of bite force (used in regular feeding habits and agonistic behaviors) is necessary to confirm or refute the studies strictly based on morphological proxies.

Empirically measuring bite force in living mammals is not a simple task. There are comparatively much more studies about skull-jaw mechanics based on morphological proxies than *in vivo* assessments. For instance, by using electrostimulation of jaw adducting muscles in anesthetized individuals, DeChow & Carlson (1983) and Thomason, Russell & Morgeli (1990) measured the bite forces of rhesus monkey (*Macaca mulata*) and Virginia opossum (*Didelphis virginiana*), respectively; Lindner et al. (1995) used transducers covered with meat-flavored rawhide to stimulate domestic dogs to bite it and act as in a tug-of-war game. Binder & Van Valkenburgh (2000) raised juvenile spotted hyenas (*Crocuta crocuta*) in captivity to assess the development of their bite force. Aguirre et al. (2002) used a transducer to measure the bite force of captured neotropical phyllostomid bats and correlate it with the niche partitioning taking into account the group phylogeny. Dumont & Herrel (2003) also used bats to describe biomechanical correlations between bite force and gape angle; these authors also highlight the lack of experimental studies addressing the *in vivo* bite force measurement in non-human mammals. Erickson, Lappin & Vliet (2003) tested the ontogeny of the bite force of alligators (*Alligator mississippiensis*) kept in a farm and compared the results with aspects of the head morphology during the individuals’ development. Bousdras et al. (2006) measured the bite force of one domestic Berkshire pig (*Sus scrofa*) through a specially designed polyethylene device molded to fit the pig’s tooth, connected to a force transducer. Brassard et al. (2020) dissected heads of 47 specimens of 26 domestic dog races, obtained bite force indexes driven from muscle and skull/jaw 3D geometric morphometrics, and used *in vivo* bite force measurements of three Belgian shepherd dogs as validation method to their biomechanical model. In a subsequent study about the domestication effects on canids, Brassard et al. (2021) dissected heads of 65 red foxes (*Vulpes vulpes*), used 3D geometric morphometrics of musculoskeletal skull/jaw system to obtain bite force, and compared with *in vivo* bite force measurements of 10 living red foxes kept in laboratory conditions.

In a rare example of a morphofunctional comprehensive study contemplating almost all aspects postulated by Wainwright & Reilly (1994), Ginot et al. (2018) managed to integrate *in vivo* bite force of wild and lab-bred murid rodents and bite force estimates through morphometric-proxies from the same individuals’ skulls as validation. The authors also integrated murid phylogenetic structure to discuss the evolution of their functional patterns. However, this approach is extremely restricted to few zoological groups that qualify for this availability of disposable individuals. Furthermore, most of these studies aiming to measure bite force *in vivo* were made using domestic and wild individuals under laboratory conditions or with individuals bred or raised in captivity. This denotes the complexity involved in collecting this kind of information, especially considering medium-sized wild mammals.

A case of study where bite force is recurrently used as a basis for niche differentiation is the coexistence of peccaries’ species, mainly between two of them: *Tayassu pecari* and *Pecari tajacu*. Kiltie (1982) was the first author to address the coexistence of these two peccary species through an ecomorphological model to explain the reduction of competition between them. According to this author, the stronger bite of the more specialist *T. pecari* allows it to feed on harder food items that are not accessible to the more generalist *P. tajacu* (Kiltie, 1982). Therefore, the synergy of these two ecomorphological arrangements prevents the competitive exclusion of one or the other species. Other independent works also detected the difference in the bite force performance between these peccary species with different methodological approaches and databases (Olmos, 1993; Sicuro & Oliveira, 2002; Hendges et al., 2019).

The ecological scenario becomes even more complex in the Brazilian Pantanal wetland (central-western Brazil) where, for at least 150 years, the feral morphotype of *S. scrofa* (“porco-monteiro”) has interacted with these two peccary species. Although the families Tayassuidae and Suidae have been phylogenetically separated since the late Eocene (Gongora, Groves & Meijaard, 2017), pigs and peccaries share several similarities in habits, diet, and general baupläne (Herring, 1972). Notwithstanding, there are key divergences in the jaw occlusion between the two families, mostly due to differences in the masseter muscle group architecture (*e.g.,* degree of pennation), molar row height in relation to the jaw condyle, and the absence of lateral movements of tayassuids’ jaw (Herring, 1972; Kiltie, 1985; Sicuro & Oliveira, 2002). On the other hand, the conspicuous nasal disc used to dig the soil to forage food items, the omnivory, and the powerful bite force used to crack roots, hard seeds, invertebrate carapaces, mollusk shells, and even bones of small vertebrates and carrion are present in both families.

The introduction of domestic pigs in the Pantanal region dates back to 1778 during the founding of Albuquerque (nowadays Corumbá, MS, Brazil). Feral hogs in Brazilian Pantanal seems to be a collateral effect of the Paraguayan War (1864–1870) when herds of pigs may have been loosened along those lowlands and proliferated favored by reduction of populations of large felids such as *Panthera onca* and *Puma concolor* heavily hunted by local settlers (Lacher, Alho & Campos, 1987; Alho & Lacher, 1991). Therefore, there is a long-term coexistence between this particular ecotype of feral hog and collared and white-lipped peccaries.

In the first paper addressing the role of ecomorphology in the interactions between sympatric peccaries and Pantanal feral hogs, Sicuro & Oliveira (2002) proposed functional measurements based on craniomandibular features to estimate the bite performance and the contribution of the jaw muscles (*i.e., temporalis* and *masseter* groups). In addition, the authors analyzed biomechanics of the head elevation muscles (*i.e.,* *semispinalis capitis*/complex muscle) related to digging habits to forage subterranean food items. In both cases, feral hogs are equally efficient or outperform peccaries in bite force and head elevation (reflected in the depth of their digging). Nevertheless, the impact in terms of food and space competition between *S. scrofa* non-domestic morphotypes (feral, wild boar, and hybrids forms) and tayassuid species in Brazil is still controversial, and it could be related to the availability of resources in the areas of occurrence (Salvador, 2017; Morais et al., 2019; Rosa, Passamani & Pompeu, 2019; Cervo & Guadagnin, 2020). In this sense, the Pantanal is a region with plenty of natural resources that could reduce most of the potential impacts of feral hogs over peccaries and even other species. However, the same may not likely happen in several other regions in Brazil where the presence of exotic *S. scrofa* in different non-domestic morphotypes (feral, wild boar, and hybrids forms from wild and domestic matrixes) became common along the XXth century (Levers, 1985; Pereira-Neto, Riet-Correa & Méndez, 1992; Sicuro, 1996; Deberdt & Scherer, 2007; Salvador, 2017; Morais et al., 2019; Cervo & Guadagnin, 2020). Therefore, understanding the jaw biomechanics can better support ecomorphological models that could explain the ecological relationships between tayassuids and non-domestic suids in Neotropics.

Hitherto, all inferences about peccaries’ skull-jaw biomechanics and its ecomorphological consequences were based on morphometric-proxies. In the last of them, Hendges et al. (2019) presented a comprehensive ecomorphological study about the three extant peccary species’ feeding biomechanics. The authors found that stronger bite forces among collared and white-lipped peccaries than Chacoan peccary (*Parachoerus wagneri*) are correlated to larger jaw-muscles attachments and shorter and more robust *corpora mandibulae*. These features are ecomorphologically coherent with the diet based on hard food items presented by *T. pecari* and *P. tajacu*. Hendges et al. (2019) also found similarities in the skull-jaw mechanics of collared and white-lipped peccaries when the analysis was size-corrected. In this same paper, the authors highlighted the importance of morphometrical proxies to assess functionality and, so far, the almost impossibility of obtaining direct bite force measurements from peccaries in the wild.

Inspired by that, we used the Brazilian Pantanal scenario–where ecomorphological mechanisms can contribute to the ecological relations between these three species–as a case study to check if the morphometric-proxies are *de facto* reliable tools to assess their bite forces. Therefore, this study aimed to empirically validate the morphometric-proxies estimates of these species’ bite force by directly measuring free-living collared and white-lipped peccaries and feral hogs’ bite force in the field for the first time. The *in vivo* measurements (in *N*) were then compared with the two independent studies that estimated the skull-jaw morphological potential of force generation based on morphometric proxies: force-indexes based on classical morphometrics by Sicuro & Oliveira (2002), and the bite force estimations based on combined geometric morphometrics and classical morphometrics by Hendges et al. (2019). The ideal experimental design for this comparison would be collecting the actual bite force of living individuals in the field and then morphometrically inferring their force from those very individuals’ skulls. This experiment, however, would be ethically and pragmatically controversial considering the species involved. Therefore, we compared these two studies with independent databases and morphometric methods with the direct bite force measurements of the wild individuals captured in the field. By doing this, we expect to validate these and other similar studies that use morphometric-proxies to assess the performance and efficiency of skull-jaw biomechanical systems.

## Materials & Methods

### Data acquisition

The wild individuals of *P. tajacu*, *T. pecari*, and feral *S. scrofa* were captured in the Brazilian Pantanal wetland, in the Nhumirim Research ranch (18°59′17″S–56°37′8.39″W), of the Empresa Brasileira de Pesquisa Agropecuária–EMBRAPA. The Nhumirim ranch is a (*c.*) 4,000 ha facility dedicated to wildlife conservation and agricultural research, and its topography represents the landscape of the Nhecolândia sub-region of Pantanal. These species live there in their habitats, keeping their ecological interactions with low-to-mild human interference.

The Brazilian Pantanal (140,000 km^2^) is situated in the upper Paraguay River in central South America (Junk et al., 2006; Bergier & Assine, 2015). It is characterized by alluvial areas under the influence of Cerrado, Amazon, and Chaco biomes (Adamoli, 1981; Alho, 2008), and the mammal biodiversity may surpass 152 species (Tomas et al., 2011). The Nhecolândia (19,661.53 km^2^) is one of 25 Pantanal sub-regions (Padovani, 2010) characterized by a seasonally flooded grassland area, with several fresh water and alkaline lakes, interspersed by a mosaic of forests and Cerrado vegetation (Padovani, 2010; Assine et al., 2016; Wilcox, 2017).

Capturing and data collecting methods were approved by the Pantanal Research Unit of EMBRAPA (CPAP-EMBRAPA) and followed the Brazilian law in force at the time, according to the Ethics Committee for Animals Use (CEUA)—Federal University of Rio de Janeiro. The CPAP-EMBRAPA also provided supporting staff and field resources to locate and capture the feral hogs and peccaries. As part of the local wildlife, the individuals had to be tracked before being captured. Once located, the individuals were lassoed and immobilized by local rangers. A total of 21 individuals were captured along 30 days of field effort: 11 feral hogs (*S. scrofa*: six adults ♀ and five adults ♂), six collared peccaries (*P. tajacu*: two adults ♀, three adults ♂, and one subadult ♂), and four white-lipped peccaries (*T. pecari*: two adults ♀, one subadult ♀ and one adult ♂). First, the individuals had their bodies measured, weighted, and GPS location collected. After that, their bite forces were tested through a mechanical device called “Bite Force Measuring Tube” (BiTu). All individuals were released after having their general condition and welfare checked.

### The Bite Force Measuring Tube (BiTu)

**Figure 1:**
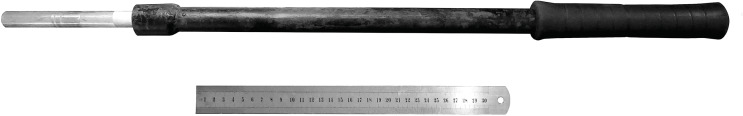
Bite Force Measuring Tube (BiTu). Overview of the Bite Force Measuring Tube (BiTu) device with an aluminum tube attached and fixed with a lateral screw in the tip of the steel rod (not visible in the image). Photo credit: Marcione B. de Oliveira.

The BiTu was designed to be used under field conditions in Pantanal by the Department of Mechanical Engineering of the Federal University of Rio de Janeiro (UFRJ) and the Technological Institute of the Pontifical Catholic University (ITUC) of Rio de Janeiro, Brazil. The BiTu is composed of a set of three aluminum tubes 150 mm long with an external diameter of 16.15 mm, with different wall thicknesses (1.6 mm, 1.8 mm, and 2.1 mm), resulting in different compression resistances. These tubes were attached to a 60 cm long rod (Fig. 1). The aluminum tubes were calibrated by an Instron Universal Testing Instrument, model 1125. Samples of each type of tube endured progressive loadings of known forces applied on a 1 cm^2^ area at the tip of the tubes to simulate the first molar’s crown upper area (M1). The choice of the M1 was based on the observation of toothrows of dozens of skulls and jaws of peccaries and feral hogs in mammal collections, which presented frequent wear of dental crowns of both lower and superior M1 (Sicuro, 1996). The constancy of this wear suggests that this is a preferred (if not optimum) physiological point to crush and grind hard food items by the three species. On the other hand, this conspicuous tooth wear flattens the tooth crown, which minimizes the influence of the dental cusps.

The calibration made by the Instron Universal Testing Instrument model 1,125 generated loading-displacement curves with elastic and plastic deformation profiles, associating the forces applied to the alterations in the tubes’ diameter. In the first phase, called elastic, the compression forces do not cause permanent deformations on the material. However, permanent alterations in the tube diameter during the plastic phase denote that the tube was submitted to forces strong enough to prompt plastic deformations. Based on the data of these loading-displacement essays, polynomial regressions (*y* =*a ± bx ± cx*^2^ ±*dx*^3^ ±*ex*^4^ ±*fx*^5^) were used to adjust the curves. After that, the lateral deformations in the bitten tubes were measured with a digital Mitutoyo caliper (Model 500–341, 150 mm, 0.01 mm), and the bite force magnitude at M1 of the individuals who managed to deform the tubes was determined through these equations. The loading-displacement curves obtained from two samples of each tube, as well as the polynomial regressions, are presented in Supplemental Information 1.

After being captured, the individuals were restrained and positioned in lateral decubitus. Then, the tip of the first tube was placed in their mouth at the M1. The natural capture stress drove some peccaries and feral hogs to actively bite the BiTu. If the tube I was easily deformed, then the following tubes were offered sequentially.

### Morphometric approach I—Sicuro & Oliveira (2002)

Sicuro & Oliveira’s (2002) original database included skulls of white-lipped peccaries (*n* = 78) and collared peccaries (*n* = 91) from several Brazilian regions and feral hogs (*n* = 11) from the Pantanal’s subregion of Nhecolândia. In the present study, we included nine new feral hogs’ skulls from the same Pantanal subregion, housed in the Mammal Collection of the Federal University of Santa Catarina (Brazil), enlarging the sample (*n* = 20).

Sicuro & Oliveira (2002) estimated the species’ bite force through a 2D lever model based on the static equilibrium equation (Σ*M* =*0*). The *temporalis* and *masseter* muscle groups’ contraction in-forces were replaced in the equation by the area of origin of these muscles’ scars at the temporal fossa and zygomatic arch, respectively. The *temporalis* in-force lever arm was the largest distance from the jaw joint to the apex of the coronoid process, while for the *masseter*, the in-force lever arm was the largest distance from the jaw joint to the angular process. The out-force lever arm was the distance from the jaw joint to the lower M1.

We used Sicuro & Oliveira’s (2002) Corrected Force-index of the Muscles Temporal and Masseter at M_1_ as an overall indicator of the final bite force of each species (CFTMM1–corrected by the Second Moment of Area that uses the measurements JHM_1_ and JWM_1_ as indicators of jawbone resistance to bending, *cf.* Sicuro & Oliveira, 2002). A complete list with the description of skull measurements and bite force-indexes equations is available on Supplemental Information 2.

### Morphometric approach II—Hendges et al. (2019)

Hendges et al. (2019) based their study on skulls of *P. tajacu* (*n* = 136), *T. pecari* (*n* = 69), and *P. wagneri* (*n* = 8) from the zoological collections of the Field Museum of Natural History and American Museum of Natural History, USA. By integrating geometric morphometric and biomechanical analyses, the authors obtained estimates of absolute bite force from craniomandibular landmarks. The equation d = $\sqrt[2]{({\mathrm{x}}_{2}-{\mathrm{x}}_{1})^{2}+({\mathrm{y}}_{2}-{\mathrm{y}}_{1})^{2}}$, where x and y are the raw coordinates for each landmark, allowed for obtaining the distance between landmarks located in specific regions of the cranium and mandible, while the size component was extracted through the generalized Procrustes analysis (GPA) as the centroid size. The centroid sizes of *temporalis* and *masseter* insertion areas were then used as proxies of muscle sizes (see Cassini & Vizcaíno, 2012). Jaw muscle torque was the masseter torque + temporalis torque. The bite force was calculated as jaw muscle torque divided by the out-lever distances at the M1. A full description of the equations used to calculate the bite force, the landmark configuration, and the in-lever and out-lever distances can be seen in Hendges et al. (2019). A complete list of landmarks is present in Supplemental Information 2.

### Statistical analyses

For comparing the three different bite force assessments, we standardized the outputs of each method (*i.e.,* CFTMM1 based on classic morphometrics, the bite force-index based on geometric morphometrics, and the *in vivo* measurements in *N* of the BiTu), centering their distributions on Z-curves. By doing this, we obtained dimensionless quantities referent to the original bite force measurements of each species according to each method. Parametric and non-parametric tests (*t*-test, Kruskal–Wallis ANOVA) were used to compare methods according to distribution issues of each case and sample sizes. The three methods to assess bite forces were statistically compared, although sample size limitations in the *in vivo* bite measurements groups hindered an overall comparison between groups/methods.

The body measurements of 19 individuals captured (the two subadult individuals were excluded to avoid unnecessary data variability) separated according to the species were compared to determine the main overall differences between groups. In addition, the biometric data of the individuals that bit the BiTu were regressed against their bite forces. Spearman correlation analyses were used to assess the relationships between the body variables and the occlusion strength. The original body measurements of all 21 individuals are available on the Supplemental Information 3. Statistical analyses were conducted in RStudio version 1.4.1106 running R version 4.1.0 (R Core Team, 2020)—packages: cairoDevice, ggplot2, ggpubr, FSA, and dunn.test) and Statistica version 8.0 (StatSoft Inc, 2007). Peccaries’ nomenclature followed Parisi Dutra et al. (2017).

## Results

### Species’ *in vivo* bite forces

Eleven out of the 21 individuals captured in the field actively bit the BiTu deforming the aluminum tubes: feral *S. scrofa n* = 7 (four adults ♀ and three adults ♂), *T. pecari n* = 2 (one adult ♀ and one adult ♂), and *P. tajacu n* = 2 (one adult ♀ and one adult ♂). Ten individuals presented a passive behavior after capture and were released after biometric information was taken; this passive behavior was interpreted as a particular response to capture stress. No pushy stimulation was made to urge these individuals to bite the BiTu; however, this impacted the sample size.

The descriptive statistics of the correspondent force according to the loading-displacement curves are presented in Table 1. The average bite forces of species are based on the maximum values on M1. The mean bite force of collared peccaries was $\bar {x}$_*P*.*tajacu*_ = 2896.4 *N* (±574.9) while the mean bite force of white-lipped peccaries was $\bar {x}$_*T*.*pecari*_ = 3336.7 *N* (±246.2). Feral hog’s mean bite force was $\bar {x}$_*S*.*scrofa*__(feral)_ = 3370.4 *N* (±930.8). The marked sexual dimorphism among the feral *S. scrofa* was reflected in their bite performance. Male feral hogs bite force mean was 3689.9 *N* (±1147.4), while female individuals achieved a mean bite force of 3130.8 *N* (±822.6).

**Table 1:** Peccaries and feral hogs *in vivo* bite forces. Peccaries and feral hogs respective bite forces measured by the BiTu. The tube columns indicate the deformation on the tube diameter in *mm* and associated force in *kgf* according to the *Instron Universal Testing Instrument* loading-displacement curves. These values were posteriorly converted to *N*.

Field No.	Tube I (*mm*)	Tube I (*kgf*)	Tube II (*mm*)	Tube II (*kgf*)	Tube III (*mm*)	Tube III (*kgf*)	Bite Force (*N*)	Species	Sex
FLS 1	0.48	283.8					2783.1	feral *S. scrofa*	f
FLS 2	0.17	259.4					2543.8	feral *S. Scrofa*	m
FLS 3	0.69	297.2					2914.5	feral *S. scrofa*	f
FLS 6	0.45	281.7	0.09	376.0			3687.3	feral *S. Scrofa*	m
FLS 8	3.70	393.4	1.04	441.1	0.06	493.4	4838.6	feral *S. scrofa*	m
FLS 15	4.38	409.7	1.06	442.1			4335.5	feral *S. scrofa*	f
FLS 21	0.11	253.9					2489.9	feral *S. Scrofa*	f
FLS 11	1.21	322.5					3162.6	*Tayassu pecari*	f
FLS 16	2.32	358.0					3510.8	*Tayassu pecari*	m
FLS 17	1.60	336.8					3302.9	*Pecari tajacu*	m
FLS 20	0.11	253.9					2489.9	*Pecari tajacu*	f

Summarized data of body measurements of 19 adult captured individuals are presented in Table 2. The three species were compared including both sexes due to the small sample sizes. The differences were significant among the three species in the five body measurements taken (K-W ANOVA results–weight: H_2,19_ = 10.63, *P* < 0.01; head length: H_2,19_ = 10.88, *P* < 0.01; body length: H_2,19_ = 13.73, *P* < 0.001; shoulder height: H_2,19_ = 10.1, *P* < 0.01; chest girth: H_2,14_ = 8.17, *P* < 0.02). Dunn’s post hoc test indicated significant weight differences only between feral *S. scrofa* and *P. tajacu* (*P* < 0.01) and *T. pecari* and *P. tajacu* (*P* = 0.04). There are marked differences in the body length (*P* < 0.001), head length (*P* < 0.01), shoulder height (*P* < 0.01), and chest girth (*P* < 0.02) between the large feral hogs and the small collared peccaries. The results denote the known smaller size of *P. tajacu* in relation to the other species (Nowak, 1999; Taber et al., 2011).

**Table 2:** Body measurements of the captured feral hogs and collared and white-lipped peccaries. Descriptive statistics of the body measurements of the feral hogs and collared and white-lipped peccaries captured in the Brazilian Pantanal of Nhecolândia, MS. Subadult individuals were not considered. The original data of all specimens are available in Supplemental Information 3.

Feral *Sus scrofa*♂	*n*	*Mean*	*Min*	*Max*	*SD*
Weight (kg)	6	62.8	30.0	100	34.9
Head length (cm)	6	37.8	30.0	43.0	4.7
Body length (cm)	6	101.8	85.0	115.0	12.6
Shoulder height (cm)	6	77.7	58.0	92.0	15.0
Chest girth (cm)	4	113.2	96.5	130.0	14.6

Feral *Sus scrofa*♀	*n*	*Mean*	*Min*	*Max*	*SD*
Weight (kg)	5	38.6	30.0	60.0	12.4
Head length (cm)	5	35.5	31.0	37.5	2.6
Body length (cm)	5	94.9	83.0	105.0	10.7
Shoulder height (cm)	5	65.8	58.0	71.5	5.1
Chest girth (cm)	3	92.5	89.0	97.5	4.4

*Tayassu pecari*	*n*	*Mean*	*Min*	*Max*	*SD*
Weight (kg)	3	38.0	32.0	43.0	5.6
Head length (cm)	3	35.7	34.5	38.0	2.0
Body length (cm)	3	80.8	78.0	83.5	2.8
Shoulder height (cm)	3	64.2	62.0	67.0	2.6
chest girth (cm)	3	95.2	88.5	99.0	5.8

*Pecari tajacu*	*n*	*Mean*	*Min*	*Max*	*SD*
Weight (kg)	5	18.5	16.0	21.0	2.3
Head length (cm)	5	27.6	25.0	29.5	1.7
Body length (cm)	5	66.7	60.0	73.5	6.1
Shoulder height (cm)	5	50.8	48.5	54.0	2.2
Chest girth (cm)	4	77.0	71.5	82.0	4.4

**Figure 2:**
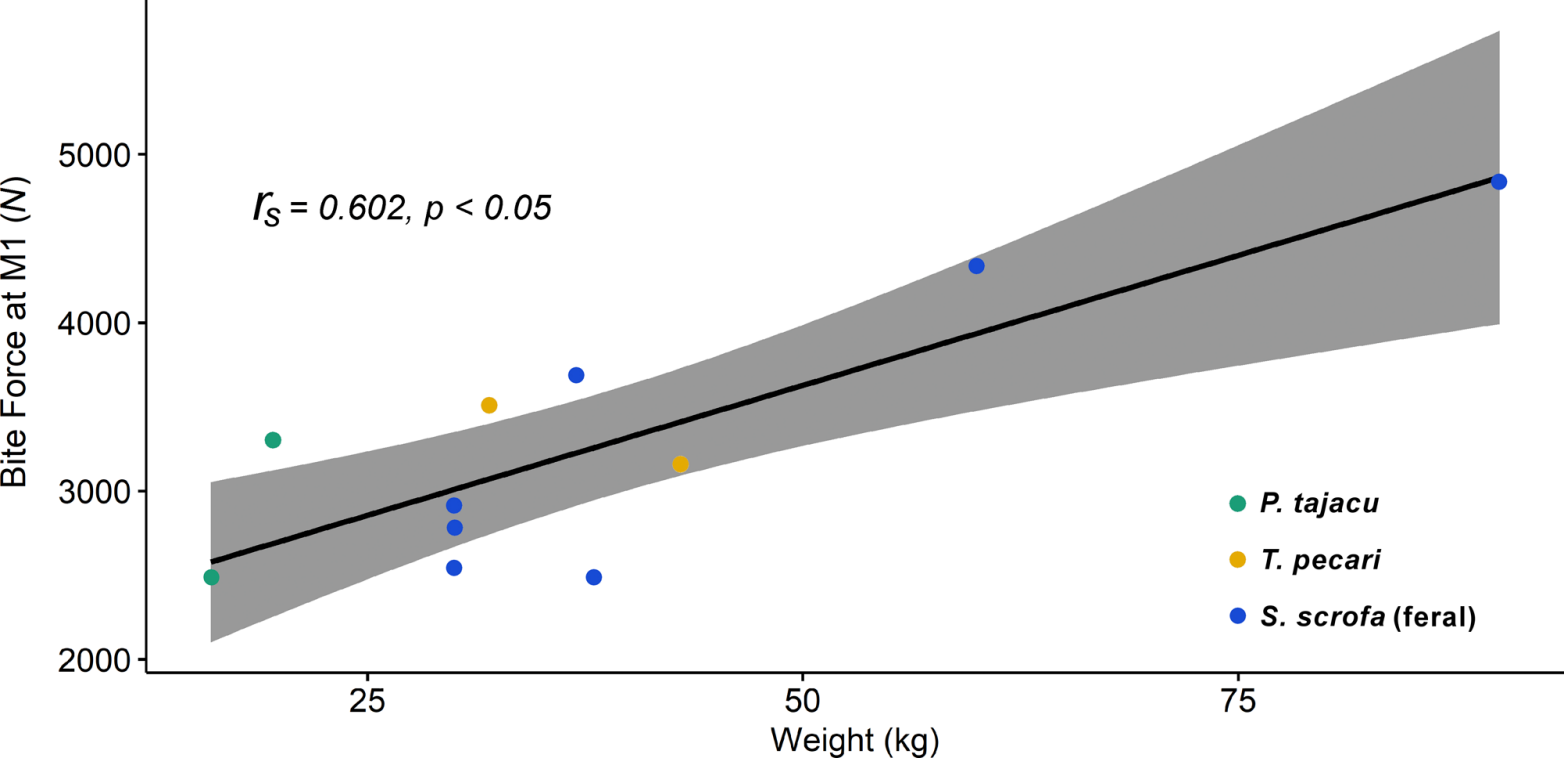
Regression of individuals’ Bite force *vs.* Body Weight and Spearman’s *r*_2_. Regression between individuals’ *in vivo* Bite Force (*N*) and their Body Weight (*kg*). The Spearman’s correlation was significant between the two measurements, denoting that heavier individuals have a more powerful bite. The Weight was the only body measurement that presented a significant correlation with the Bite Force.

The Spearman correlation between the *in vivo* bite force outputs and body measurements of the same individuals of the three species (*n* = 11, except to chest girth *n* = 8) presented significance (r_s_: 0.60, *p* < 0.049) only between the bite force (*N*) and individuals’ weight (*kg*) - Fig. 2. We found no correlation between the bite force (*N*) and the head length (cm), despite the marked head size difference between *P. tajacu* and feral *S. scrofa* (r_s_: 0.49, *p* = 0.17). It may suggest a more efficient skull-jaw biomechanical system of the collared peccaries to generate strong bite forces at the M1 over the feral hogs’ skull-jaw arrangement. Sicuro & Oliveira (2002) had already highlighted the efficiency of peccaries’ skull pattern to produce strong bite forces according to their size compared with feral *S. scrofa*’s skull.

### Bite force-indexes comparisons

The mean values of Sicuro & Oliveira’s (2002) index CRTMM1 representing the overall bite force at the M1 were, respectively: $\bar {x}$_*P*.*tajacu*_ = 16.85 (±1.18); $\bar {x}$_*T*.*pecari*_ = 20.51 (±1.35); and $\bar {x}$_*S*.*scrofa*__(feral)_ = 21.56 (±2.11). These values are not “force” in the sense of *N* units but a morphofunctional potential of force generation. The K-W ANOVA indicated differences between the three species (*H*_2,189_ = 132.04, *P* < 0.0001). Significant differences were observed between *P. tajacu* and *T. pecari* (Dunn’s post hoc test; *P* < 0.0001) and *P. tajacu* and feral *S. scrofa* (Dunn’s post hoc test; *P* < 0.0001); however, no significant difference was found between *T. pecari* and feral *S. scrofa* (*P* > 0.71).

Based on Hendges et al. (2019) geometric morphometrics-proxies, the mean value of the collared peccary’s bite force-index at M1 was $\bar {x}$_*P*.*tajacu*_ = 10.70 (±0.77), and the mean for the white-lipped peccary’s $\bar {x}$_*T*.*pecari*_ = 13.24 (±0.75). The difference between the peccary species was significant (t-test: t_1,139.7_ = −22.76, *p* < 0.00001).

The comparison between peccaries’ bite force standardized values, based on the two morphometric methods (classic and geometric), indicate significant differences between groups (*H*_4,394_ = 271.61, *P* < 0.0001). No significant differences were found in the bite force estimates between the two morphometric-proxies-based methods for *P. tajacu* (Dunn’s post hoc test; *P* = 0.41) and for *T. pecari* (Dunn’s post hoc test; *P* = 0.13). There were significant differences between *P. tajacu*’s bite force estimates and *T. pecari* (Dunn’s post hoc test; *P* < 0.00001), and feral *S. scrofa* (Dunn’s post hoc test; *P* < 0.00001). However, no significant differences were found between the bite force-indexes of *T. pecari* and those of feral *S. scrofa* in both classical and geometric morphometrics approaches (Dunn’s post hoc test; *P* = 1.00). These results denote an overall similarity between the bite force estimates and relations between the species, despite the different morphometric methods and databases.

### *In vivo* bite forces *vs.* bite force estimates

**Figure 3:**
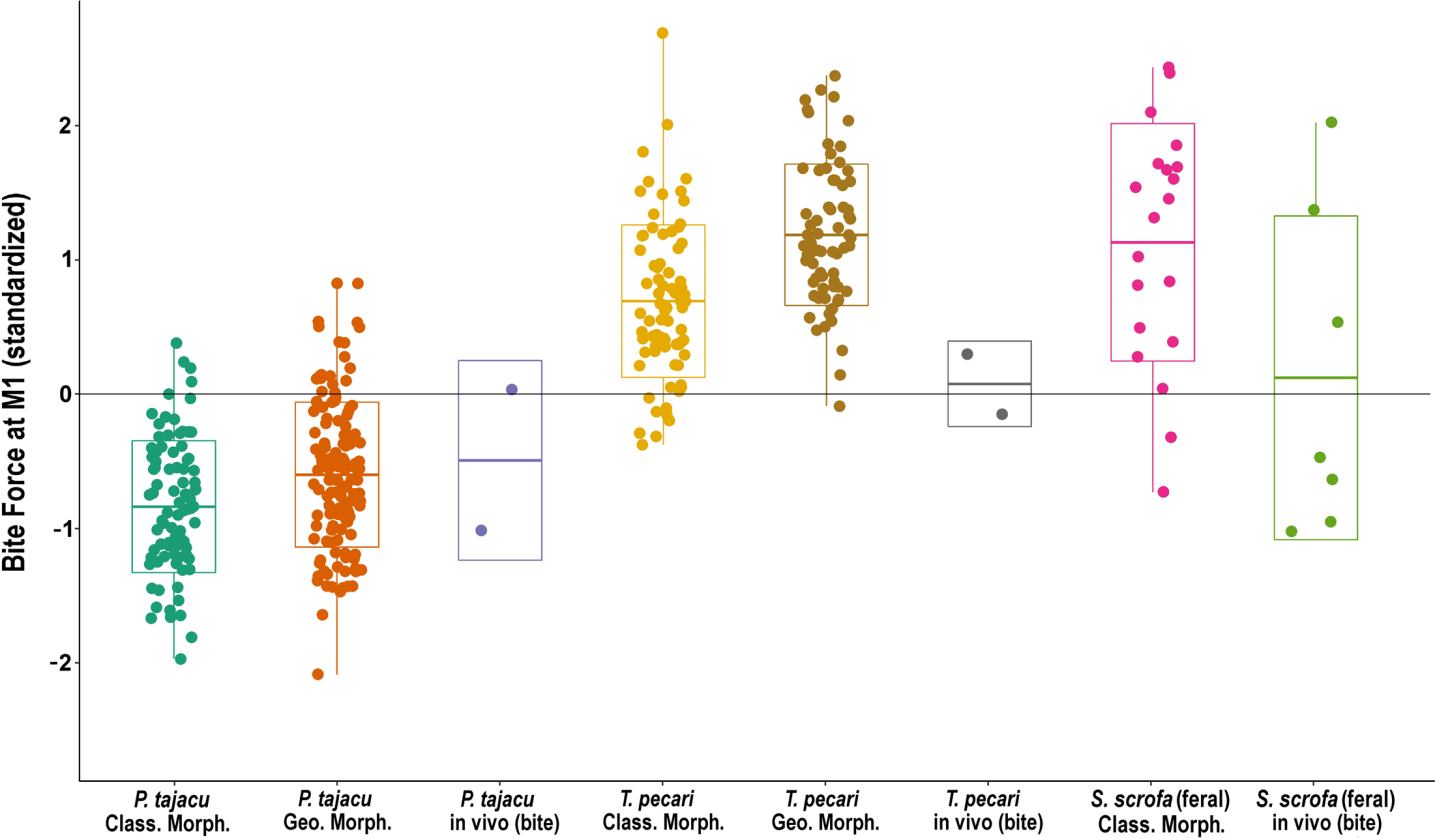
Box-plot with the standardized values of bite forces estimated by the two morphometric-proxies methods and the *in vivo* measurements, according to the species. Box-plot with jitter-function of the standardized values of each bite force assessment method. Bite force estimates based on Sicuro & Oliveira, 2002 Classic Morphometrics are indicated as Class.Morph.; Hendges et al. (2019) bite force estimates using Geometric Morphometrics are denoted as Geo.Morph.; and the actual bite force measurements are indicated as *in vivo*. (Legend: bar = mean, box = SD, whiskers = max/min).

The standardized data of the bite force-indexes driven from classic and geometric morphometrics approaches and bite force results empirically acquired in the field are presented in Fig. 3. The sample size limitations hindered further inferential statistical analyses, including the *in vivo* results. Nevertheless, there are similarities between the morphometric-proxies bite force estimates and the empirical measurement of bite force on the living wild individuals, at least at the limits of their frequency distributions. The *in vivo* bite forces measured in the male and female individuals of *P. tajacu* followed the same trends of the inferred bite force of both morphometric-based methods. The *T. pecari*’s actual bite force was below the mid-range of the morphometric-proxies estimates, but the correspondence with the force-indexes is still pretty valid, taking into account the small sample.

Even considering the limited sample, the *in vivo* measurements indicate that white-lipped peccaries’ real bite is stronger than that of the collared peccaries. The results of the estimated and performed bite force of the Pantanal feral hogs presented marked differences according to the mean values. However, both methods denoted similar variation in the Pantanal feral hog’s bite forces. It is important to stress that, diversely of the two peccary species, there is a marked sexual dimorphism among suids (including the Pantanal feral hogs), and that may be contributing to this wide variation. Notwithstanding the differences in databases and morphometric approaches and the limited sample of individuals in the *in vivo* experiment, the outputs showed a good correspondence.

## Discussion

**Figure 4:**
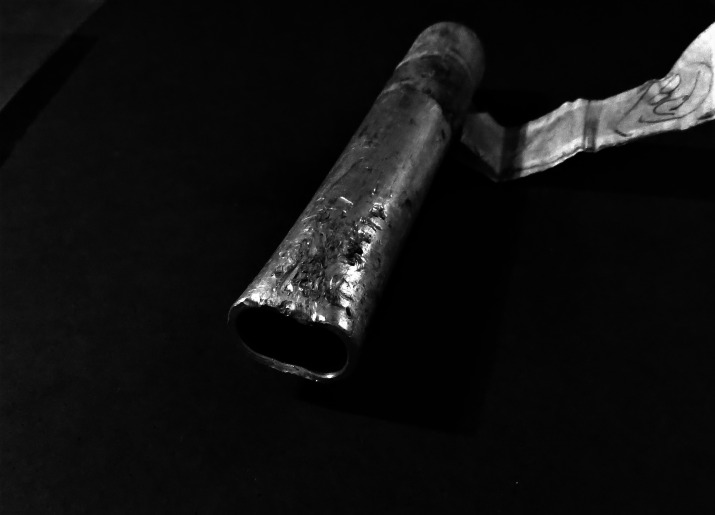
BiTu tube deformed after being bitten. The aspect of a tube deformed after a feral hog bite. There is a clear alteration of the tube’s original diameter. The difference between the original external diameter and the deformation denotes the displacement corresponding to a compression force. The tooth marks on the tube’s surface were not considered and only the full effect of the jaw occlusion was registered. The tubes were tagged for posterior measurement and museum cataloging. Photo credit: Marcione B. de Oliveira.

We presented for the first time the actual magnitude of the bite forces of wild collared and white-lipped peccaries and feral hogs in their own habitat in the Brazilian Pantanal. The impact of non-domestic pig morphotypes is a recurrent issue in several places worldwide, particularly in Brazil, where they are in several biomes (*e.g.*, Ilse & Hellgren, 1995; Gabor & Hellgren, 2000; Salvador, 2017; Cervo & Guadagnin, 2020; Doutel-Ribas et al., 2019). Although the main aim of the present study was not to discuss the ecological implications of bite force differences among those species (this subject was already addressed by our team and some other authors in previous works), the *in vivo* results of the species’ bite force in the field validated those morphometric-proxies-based assessments. The BiTu proved to be a practical and low-cost way to get *in vivo* bite force information. Furthermore, its principle could be adjusted to fit other zoological groups. It was designed to match field demands on robustness, practicability, and versatility, although some crudity of the measurements (*cf.* Sun et al., 2015, fig. 5). For instance, eventual tooth marks dug on the aluminum tubes were not accounted as “deformations”, and only alterations on the tubes’ diameter were registered as effective bites (Fig. 4). On the other hand, using only the deformations on tubes’ diameter is an unequivocal register of the overall bite force at M1, despite particularities on the teeth crown.

However, *in vivo* bite measurements are dependent on several factors, such as the species, sex, age class, bite context, stress, skull-jaw morphology, bite positioning relative to the jaw joint, gape angle, the method used, and so on. It is also important to highlight that when one measures the bite force of an animal, this measurement indicates this individual could exert at least that bite force. As we can tell based on our fieldwork, there are several reasons why captured individuals may be biting using much less force than they could, including the fact that they simply are not willing to bite at all. Despite our success in capturing 21 medium-sized wild mammals in 30 days (among dozens of others that escaped), we have seen our sample size reduced by almost half simply because individuals did not bite the device or did so lightly that nor did they deform the tubes. On the other hand, the individuals that actively bit the BiTu did it with an unequivocal aggressiveness.

Therefore, the *in vivo* measurements should be evaluated in a comparative context, ideally with identical methods and protocols. For instance, Erickson, Lappin & Vliet (2003) comparing bite forces of several groups indicate *Homo sapiens*’ bite force reaching 700 *N* (or 800 *N*, according to Van Eijden, 1991), while *Pongo pygmaeus*’ bites may reach almost 1,900 *N*. Binder & Van Valkenburgh (2000) registered a young spotted hyena’s (c. 2.5-years-old) bite of 1,300 *N* at incisors, *i.e.,* the longest jaw’s resistance moment arm, and an adult spotted hyena’s bite over 4,500 *N*. Thus, if an adult hyena’s incisors bite may reach this magnitude, it will be much larger at the carnassials where jaw’s resistance moment arm is pretty shorter.

*In vivo* bite force measurements around 3,000 *N* at M1 may seem overestimated, considering peccaries and feral hogs. However, it is worth remembering that these species are omnivorous, and Pereira-Neto, Riet-Correa & Méndez (1992) describe problems related to exotic wild boars, feral hogs, and hybrids forms in Southern Brazil and Uruguay attacking crops and killing and feeding on sheep herds. During our visits to Pantanal, we collected several anecdotic reports of conflicts involving feral hogs and the local people. Among them, one caught our attention for describing an accident with a local “pantaneiro-man” during the capture large feral hog female. After lassoing her, he got off his horse and, when trying to immobilize her by grabbing her ear, he missed his grip, and his four fingers went into the sow’s mouth and were severed with a single bite. In another context, Bousdras et al. (2006), using implanted transducers in casted premolars, detected domestic Berkshire pigs’ bite forces around 560 *N* during the normal chewing activities with soft silicon elements like normal food consistency. It means that those domestic pigs–doing nothing else than chewing soft food–performed bite forces up to 560 *N*. Therefore, the high bite force values we measured here with wild stressed individuals in defense context are likely near to their maximum bite forces.

Another source of variation to the bite performance is the marked sexual dimorphism in Suidae species. Sicuro, Neves & Oliveira (2011) found few significant differences in the skull morphology between male and female peccaries (*P. tajacu* and *T. pecari*), but no significant sexual-related differences in their bite force-indexes. However, the feral *S. scrofa*’s marked sexual dimorphism seems to significantly contribute to the data variability to the bite force-indexes and *in vivo* measurements.

One could also argue that the impact of the capture stress on the bite force output, and even if the bite we measured has no physiological use on feeding. Notwithstanding, all empirical bite force studies cited here also face practical or even methodological constraints. Most of them used individuals that were bred, raised, or held in captivity, precisely because of the several difficulties to collect biomechanical information from wild free-ranging individuals (*e.g.*, DeChow & Carlson, 1983; Thomason, Russell & Morgeli, 1990; Lindner et al., 1995; Binder & Van Valkenburgh, 2000; Erickson, Lappin & Vliet, 2003; Bousdras et al., 2006). On this matter, Sicuro (2006) indicated marked bone alterations on the skulls of specimens of different Felidae species from zoos. The author has excluded several skulls of *Neofelis nebulosa* and *Panthera pardus* individuals from his database due to aberrant bone crests around the orbits and malar bone, bone fragility, or signs of osteopathy, probably related to their life, lack of activity, or diet in captivity. Therefore, functional studies based on zoo individuals could be influenced by possible captivity-driven variations in the musculoskeletal systems. Furthermore, other empirical studies assessed bite measurements by artificially inducing muscle contractions by electrostimulation on anesthetized individuals (*e.g.*, DeChow & Carlson, 1983; Thomason, Russell & Morgeli, 1990; Lindner et al., 1995). Exceptions can be cited for using wild specimens captured in the field, such as Aguirre et al. (2002) and Dumont & Herrel (2003) with bats, Ginot et al. (2018) with murid rodents, and Brassard et al. (2021) with red foxes.

Far from criticizing these pertinent studies, we aim to stress the difficulty of assessing reliable information about the mammals’ bite force, let alone the wild medium-sized ones. All these works brought factual information about the bite force magnitude on groups whose skull-jaw mechanics are usually approached through morphometric-proxies. Ultimately, *in vivo* measurements indicate that the individuals can at least perform a given bite force in specific behavioral displays (*e.g.*, feeding or fighting). Accordingly, our data indicate that despite eventual biases in *in vivo* measurements and proxy-based estimations, the results are fairly similar.

Nevertheless, the morphometric-proxies-based methods using the static equilibrium equation assume that the out-force is the maximum that a given skull-jaw pattern can produce. That said, this kind of study only makes sense for comparisons between species or, at least, different morphotypes (since they share morphological similarities and phylogenetic relations).

In the present study, we took advantage of the databases from Sicuro & Oliveira (2002) and Hendges et al. (2019) to test correspondences between two morphofunctional proxies-based approaches and *in vivo* measurement of bite force. These two studies dealt with the same subject and focused on the tayassuids/suids skull-jaw mechanics and ecomorphology. Therefore, the similarity we found between the force indexes obtained through these two studies denotes their biomechanical analyses’ consistency. The fact that the *in vivo* measurements indicated bite forces with similar trends observed in the two-independent morphometric studies corroborates the thesis that the linear and geometric morphometrics-proxies approaches detected a consistent biomechanical signal. Moreover, Sicuro & Oliveira’s (2002) morphofunctional analyses already indicated the superiority of peccaries’ skull pattern to produce strong bite forces according to their size compared with feral hog’s skull. The absence of correlation between the bite force (*N*) and the head length (cm), although the evident difference between *P. tajacu* and feral *S. scrofa* head-sizes, suggests that–at least–the collared peccaries present a more efficient skull-jaw biomechanical system to generate strong bite forces at the M1 than the feral hogs.

The results assessed through the BiTu were also consistent with the conclusions of other authors such as Kiltie (1982) and Olmos (1993), where collared peccaries present a weaker bite than white-lipped peccaries. Despite the difficulties in collecting the data directly from wild specimens in the field, this is the best empirical validation of a skull morphofunctional study with peccaries and feral hogs hitherto. Accordingly, not only the superior bite force of white-lipped peccaries and feral hogs over the collared peccaries was evinced, but also the overall similarity between *T. pecari* and the Pantanal feral *S. scrofa* bite performances.

Ultimately, the results support the idea that morphometric methods imbued with relevant anatomical information can provide meaningful information about the real-life functionality of organisms’ musculoskeletal structures. Therefore, our results allowed quantifying an important biomechanical feature that brings a far more reliable baseline to discuss the relations between coexisting peccary species and feral hogs based on ecomorphological models.

## Conclusions

The *in vivo* results of peccaries and feral hogs’ bite forces supported the biomechanical inferences based on morphometric proxies in previous works. Namely, the analyses indicate** similar bite forces in white-lipped peccaries and Pantanal feral hogs and their superiority over collared peccaries’ bite force. The bite performances predicted by the previous morphometric-proxies models presented a clear correspondence with the *in vivo* experiments. The methods used on the two morphometric-proxies studies compared in the present work followed a long lineage of biomechanical studies and morphofunctional approaches. Their assumptions (in some different and more complex ways) are present in several morphofunctional studies.

Thus, besides endorsing the validity of the morphometric-proxies studies analyzed here, the present study also highlights the importance of *in vivo* biomechanical studies to support the morphofunctional models in general. Despite the complexity of the methods used to address biomechanical systems, there is always a gap between the functional inferences and the actual responses of these biomechanical complexes in living organisms. The relatively few works evaluating *in vivo* musculoskeletal performances are a valuable epistemological assurance–despite all methodological constraints involved–to several other morphometric-proxies-based studies and, consequently, to their ecological and evolutionary inferences, as well.

## Supplemental Information

10.7717/peerj.11948/supp-1 Supplemental Information 1 BiTu loading-displacement curves Tubes I, II, and III loading-displacement curves and respective the polynomial regressions obtained from two samples of each tube by the Instron Universal Testing Instrument, model 1125 with its original outputs in *kgf*. Conversions to *N* used in the study were based on the factor 1 *kgf* = 9.80665 *N*.

10.7717/peerj.11948/supp-2 Supplemental Information 2 *Sicuro & Oliveira*’s *(2002)* and Hendges et al. (2019) skull measurements, force-indexes, landmarks, and acronyms *Sicuro & Oliveira*’s *(2002)* and *Hendges et al. (2019)* skull measurements, force-indexes, landmarks, and acronyms Full list of *Sicuro and Oliveira*’s *(2002)* skull measurements, force-indexes, and acronyms. Definition of landmarks and semi-landmarks placed on the cranium and mandible of each peccary specimen in Hendges et al. (2019). Descriptions of the formula for each biomechanical variable estimated for peccary species in *Hendges et al. (2019)*. * = multiplied by; / = dividing by**.**

10.7717/peerj.11948/supp-3 Supplemental Information 3 Body measurements of the 21 individuals captured in the Brazilian Pantanal of Nhecolândia Complete matrix with the original body measurements of the 21 individuals captured in the Brazilian Pantanal of Nhecolândia and identified by their field numbers. Subadult individuals’ measurements presented here were not included in the analyses.

10.7717/peerj.11948/supp-4 Supplemental Information 4 Raw Data

## Additional Information and Declarations

### Data Availability

The following information was supplied regarding data availability: The raw measurements are available in the Supplemental Files.
