# Supplementary material for: Quantifying bite force in coexisting tayassuids and feral suids: a comparison between morphometric functional proxies and in vivo measurements
Source: PeerJ. 2021 Aug 12;9:e11948. doi: 10.7717/peerj.11948 (PMC8364746; doi:10.7717/peerj.11948)

Supplement 1

Tubes I, II, and III loading-displacement curves and respective the polynomial regressions obtained from two samples of each tube by the Instron Universal Testing Instrument, model 1125 with its original outputs in *kgf*. Conversions to *N* used in the study were based on the factor 1 *kgf* = 9.80665 *N*.


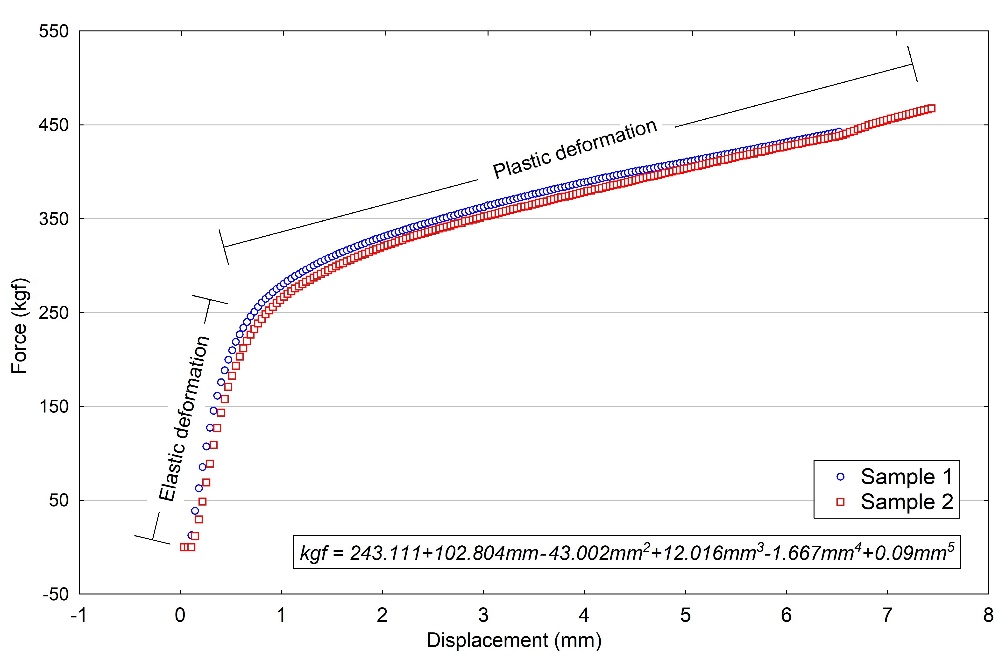


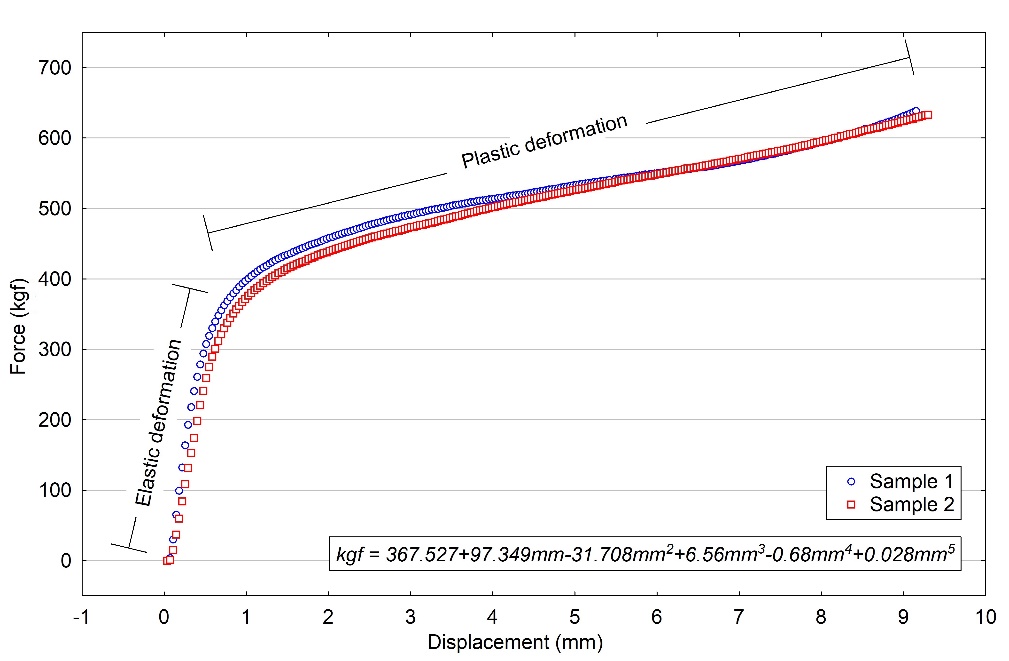


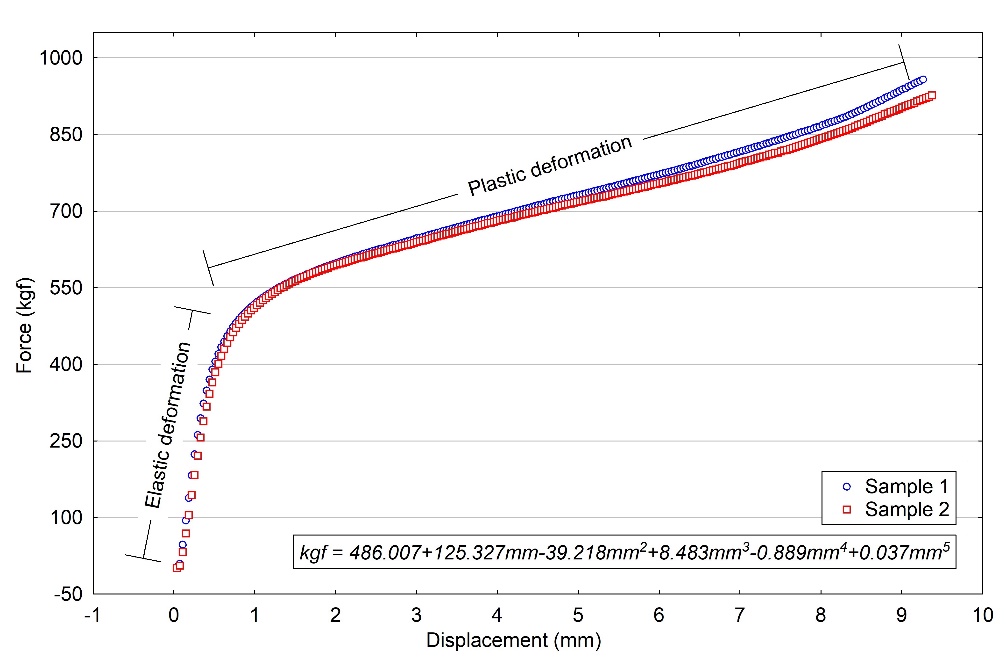

Supplement: Supplemental Information 1 — Tubes I, II, and III loading-displacement curves and respective the polynomial regressions obtained from two samples of each tube by the Instron Universal Testing Instrument, model 1125 with its original outputs in kgf. Conversions to N used in the study were based on the factor 1 kgf = 9.80665 N. [file peerj-09-11948-s001.docx]
