# Supplementary material for: Quantifying bite force in coexisting tayassuids and feral suids: a comparison between morphometric functional proxies and in vivo measurements
Source: PeerJ. 2021 Aug 12;9:e11948. doi: 10.7717/peerj.11948 (PMC8364746; doi:10.7717/peerj.11948)
Supplement: Supplemental Information 2 — Sicuro & Oliveira’s (2002) and Hendges et al. (2019) skull measurements, force-indexes, landmarks, and acronyms Full list of Sicuro and Oliveira’s (2002) skull measurements, force-indexes, and acronyms. Definition of landmarks and semi-landmarks placed on the cranium and mandible of each peccary specimen in Hendges et al. (2019). Descriptions of the formula for each biomechanical variable estimated for peccary species in Hendges et al. (2019). * = multiplied by; / = dividing by. [file peerj-09-11948-s002.docx]

Supplement 2

Full list of Sicuro and Oliveira’s (2002) skull measurements, force-indexes, and acronyms.

| BCL | Basal condyle length |
| --- | --- |
| CCL | Condyle to canine length of jaw |
| CM_1_L | Condyle to M_1_ length of jaw |
| CSW | complex muscle scar width |
| JHM_1_ | Jaw height at M_1_ |
| JWM_1_ | Jaw width at M_1_ |
| LCW | lambdoidal crest width |
| MMA | Masseter muscle moment arm |
| MSL | Masseter muscle scar length |
| MSW | Masseter muscle scar width |
| ORH | Occipital region height |
| PTW | Posttemporal fossa width |
| RWP^2^ | Rostral width at the 2^nd^ premolar P^2^ |
| SL | Skull length |
| TFL | Temporal fossa length |
| TMA | Temporal muscle moment arm |
| ZIW | Zygomatic arches internal width |

| SMA | $\sqrt[4]{\frac{\pi\times\frac{{JWM}_{1}}{2}\times\left( \frac{{JHM}_{1}}{2} \right)^{3}}{4}}$ |
| --- | --- |
| TMW | $\frac{\left( ZIB- \frac{BBC+POC}{2} \right)}{2}$ |
| FTM1 | $\frac{\sqrt[2]{TFL\times TMW}\times TMA}{{CM}_{1}L}$ |
| FMM1 | $\frac{\sqrt[3]{MSL\times MSW\times MMA}\times MMA}{{CM}_{1}L}$ |
| FTMM1 | $\sqrt[2]{{FMM}_{1}\times{FTM}_{1}}$ |
| CFTMM1 | $\sqrt[2]{SMA\times{FTMM}_{1}}$ |

Definition of landmarks and semi-landmarks placed on the cranium and mandible of each peccary specimen in Hendges et al., 2019.

*Ventral view of the cranium:*

**L1** = midpoint of central incisors; **L2** = posteriormost point of interpalatine suture; **L3** = vertical projections of landmark 6 and 7 in an angle of 90 degrees; **L4** = anteriormost point of the foramen magnum along the midline; **L5** = posteriormost margin of the occipital condyle; **L6** = anteriormost external border of the auditory bulla; **L7** = lateral point of the skull at the region of post-glenoid process; **L8** = suture between jugal and squamosal in the zygomatic arch; **L9, 11, 12, 13, 15, 16, 33** = temporal muscle insertion area; **L10** = curvature between the maxilla and the zygomatic arch**; L14** = extremity of the post-glenoid process; **L17, 18, 19, 20** = third molar area; **L21, 22, 23, 24** = second molar area; **L25, 26, 27, 28** = first molar area; **L29** = anteriormost tip of the first premolar; **L30** = posteriormost point of the lateral canine; **L31** = anteriormost point of the lateral canine; **L32** = posteriormost point of the lateral incisor.

*Lateral view of the mandible*:

**L1** = posteriormost point of the canine alveolus; **L2** = midpoint of the diastema **L3** = anteriormost point of the first premolar alveolus; **L4** = anteriormost point of the first molar alveolus; **L5** = junction between ramus and mandibular corpora at third molar; **L6** = anteriormost point of the coronoid process; **L7** = midpoint of the coronoid process; **L8 =** tip of the coronoid process; **L9** = posteriormost point of the coronoid process; **L10** = point of maximum curvature between the coronoid and condylar process; **L11** = tip of the condylar process as seen in the lateral view; **L12** = end of the lateral border of the angular process; **L13** = junction between mandibular corpora and masseteric crest; **L14, 15, 16** = vertical projections of landmark 4, 3 and 2 (respectively) in an angle of 90 degrees; **L17**: lateral cavity in mandibular corpora at the region of canine as seen in the lateral view, **L18** = anteriormost point of the incisor alveolus; **L19** = anteriomost point of the canine alveolus; **L20** = midpoint of the canine alveolus.

Descriptions of the formula for each biomechanical variable estimated for peccary species in Hendges et al., 2019. * = multiplied by; / = dividing by**.**

| Biomechanical variables | Formula |
| --- | --- |
| Masseter torque | masseter centroid size * the in-lever distance from the tip of the condylar process (L11) to the junction between the ramus and mandibular corpus, at M3 (L5) |
| Temporalis torque | temporalis centroid size * in-lever distance from the tip of the post-glenoid process (L14) to the curvature between the maxilla and the zygomatic arch (L10). |
| Jaw muscle torque | masseter torque + temporalis torque |
| Bite force | jaw muscle torque / out-lever distances at incisor (L11 to L18), canine (L11 to L1), first premolar (L11 to L3), and first molar (L11 to L4). |
| Bite stress at molars | bite force at the first molar / centroid size of M1, M2 and M3 |
| Resistance to bending | corpus height dividing by mandible length (distance from L11 to L18) |
| Stress under bending | torque at the incisors (FB * L11-L1) / corpus height |
| Resistance to shear | corpus width * corpus height * π |
| Shear stress | bite force at M1 / corpus width * corpus height * π |
| Condylar reaction force | jaw elevator torque at M1 / out-lever distance from the tip of the condylar process (L11) to the anteriormost point of the first molar alveolus (L4). |
| Condyle stress | condyle reaction force / condylar area (= condyle length * condyle width) |
| Relative symphysis length | symphysis length / mandible length. |
