# Supplementary material for: Quantifying bite force in coexisting tayassuids and feral suids: a comparison between morphometric functional proxies and in vivo measurements
Source: PeerJ. 2021 Aug 12;9:e11948. doi: 10.7717/peerj.11948 (PMC8364746; doi:10.7717/peerj.11948)
Supplement: Supplemental Information 3 — Complete matrix with the original body measurements of the 21 individuals captured in the Brazilian Pantanal of Nhecolândia and identified by their field numbers. Subadult individuals’ measurements presented here were not included in the analyses. [file peerj-09-11948-s003.docx]

Supplement 3

Original body measurements of captured individuals identified by their field numbers. Subadult individuals’ measurements were not included in the analyses.

|  | Species | Sex | Age-class | Weight (kg) | Head length (cm) | Body length (cm) | Shoulder height (cm) | Chest girth (cm) | Max. Bite force (N) |
| --- | --- | --- | --- | --- | --- | --- | --- | --- | --- |
| FLS 1 | *S. Scrofa (feral)* | f | adult | 30.0 | 37.0 | 105.0 | 67.0 |  | 2783.1 |
| FLS 3 | *S. Scrofa (feral)* | f | adult | 30.0 | 36.0 | 97.0 | 58.0 |  | 2914.5 |
| FLS 9 | *S. Scrofa (feral)* | f | adult | 35.0 | 31.0 | 83.0 | 64.0 | 89.0 |  |
| FLS 15 | *S. Scrofa (feral)* | f | adult | 60.0 | 37.5 | 105.0 | 71.5 | 97.5 | 4335.5 |
| FLS 21 | *S. Scrofa (feral)* | f | adult | 38.0 | 36.0 | 84.5 | 68.5 | 91.0 | 2489.9 |
| FLS 2 | *S. Scrofa (feral)* | m | adult | 30.0 | 36.0 | 90.0 | 62.0 |  | 2543.8 |
| FLS 5 | *S. Scrofa (feral)* | m | adult | 30.0 | 30.0 | 85.0 | 58.0 |  |  |
| FLS 6 | *S. Scrofa (feral)* | m | adult | 37.0 | 36.0 | 98.0 | 75.0 | 96.5 | 3687.3 |
| FLS 7 | *S. Scrofa (feral)* | m | adult | 80.0 | 41.0 | 111.0 | 90.0 | 107.0 |  |
| FLS 8 | *S. Scrofa (feral)* | m | adult | 90.0 | 43.0 | 112.0 | 89.0 | 119.5 | 4838.6 |
| FLS 10 | *S. Scrofa (feral)* | m | adult | 110.0 | 40.5 | 115.0 | 92.0 | 130.0 |  |
| FLS 11 | *T. pecari* | f | adult | 43.0 | 38.0 | 81.0 | 67.0 | 99.0 | 3162.6 |
| FLS 12 | *T. pecari* | f | subadult* | 6.0 | 21.5 | 43.0 | 38.5 | 54.5 |  |
| FLS 14 | *T. pecari* | f | adult | 39.0 | 34.5 | 83.5 | 62.0 | 98.0 |  |
| FLS 16 | *T. pecari* | m | adult | 32.0 | 34.5 | 78.0 | 63.5 | 88.5 | 3510.8 |
| FLS 4 | *P. tajacu* | m | adult | 20.0 | 25.0 | 61.0 | 50.0 |  |  |
| FLS 18 | *P. tajacu* | f | adult | 21.0 | 28.5 | 71.5 | 52.0 | 76.0 |  |
| FLS 20 | *P. tajacu* | f | adult | 16.0 | 28.0 | 73.5 | 49.5 | 82.0 | 2489.9 |
| FLS 13 | *P. tajacu* | m | adult | 16.0 | 27.0 | 60.0 | 48.5 | 71.5 |  |
| FLS 17 | *P. tajacu* | m | adult | 19.5 | 29.5 | 67.5 | 54.0 | 78.5 | 3302.9 |
| FLS 19 | *P. tajacu* | m | subadult* | 8.0 | 22.0 | 51.0 | 40.0 | 46.5 |  |
